# Novel Immune Cell Subsets Exhibit Different Associations With Vascular Outcomes in Chronic Kidney Disease Patients—Identifying Potential Biomarkers

**DOI:** 10.3389/fmed.2021.618286

**Published:** 2021-05-25

**Authors:** Javier Rodríguez-Carrio, Natalia Carrillo-López, Catalina Ulloa, Beatriz Martín-Carro, Carmen Rodríguez-Suárez, Manuel Naves-Díaz, Emilio Sánchez-Álvarez, Minerva Rodríguez-García, Maria Vittoria Arcidiacono, Belinda Fernández-Mariño, Jorge B. Cannata-Andía, Ana Suárez, Adriana S. Dusso

**Affiliations:** ^1^Area of Immunology, Department of Functional Biology, University of Oviedo, Oviedo, Spain; ^2^Bone and Mineral Research Unit, Hospital Universitario Central de Asturias, Oviedo, Spain; ^3^Instituto de Investigación Sanitaria del Principado de Asturias, Oviedo, Spain; ^4^REDinREN-ISCIII, Oviedo, Spain; ^5^Department of Nephrology, Hospital Universitario de Cabueñes, Gijón, Spain; ^6^Division of Nephrology, Hospital Universitario Central de Asturias, Oviedo, Spain; ^7^Department of Radiology, Hospital Universitario Central de Asturias, Oviedo, Spain; ^8^Department of Medicine, University of Oviedo, Oviedo, Spain; ^9^Division of Endocrinology, Metabolism and Lipid Research, Washington University School of Medicine, St. Louis, MO, United States

**Keywords:** atherosclerosis, vasa vasorum, CKD, inflammation, vascular outcomes

## Abstract

**Background and Aims:** Alterations in novel immune cell subsets, such as angiogenic T cells (Tang), senescent T cells (CD4^+^CD28^null^), and monocyte subsets are associated with impaired vascular homeostasis in several inflammatory conditions. However, mediators underlying vascular deterioration in chronic kidney disease (CKD) are poorly characterized. This study assessed their role in the vascular deterioration of CKD using a broad spectrum of surrogate markers ranging from altered functionality to overt calcification.

**Methods:** Tang (CD3^+^CD31^+^CXCR4^+^), CD4^+^CD28^null^ cells, and monocytes [CD14/CD16 subsets and angiotensin-converting enzyme (ACE) expression] were measured in peripheral blood by flow cytometry in 33 CKD stage 5 patients undergoing peritoneal dialysis (CKD5-PD) and 15 healthy controls (HCs). Analyses were replicated in a hemodialysis cohort. Vascular surrogate markers (including adventitial vasa vasorum, pulse wave velocity, intima-media thickness, and vascular calcification) were assessed by appropriate imaging methods.

**Results:** In CKD5-PD, decreased Tang levels (*p* < 0.001) were unrelated to clinical features or traditional cardiovascular (CV) risk factors but correlated negatively with troponin T levels (*r* = −0.550, *p* = 0.003). Instead, CD4^+^CD28^null^ frequency was increased (*p* < 0.001), especially in those with vascular calcifications. Quantitative and qualitative differences were also observed within the monocyte pool, a shift toward CD16^+^ subsets and ACE expression being found in CKD. Equivalent results were observed in the replication cohort. Each subset associated distinctly with adverse vascular outcomes in univariate and multivariate analyses: while Tang depletion was linked to poor vascular function and subclinical atherosclerosis, increases in CD4^+^CD28^null^ were associated with overt vascular thickening and calcification. Monocytes were not independently associated with vascular outcomes in CKD patients.

**Conclusions:** Novel T cell and monocyte subsets are altered in CKD. Altered T-cell subpopulations, but not monocytes, exhibited distinct associations with different vascular outcomes in CKD. Tang are emerging biomarkers of subclinical vascular deterioration in CKD.

## Introduction

Chronic kidney disease (CKD) is hallmarked by chronic systemic inflammation, which not only plays a role in aggravating renal damage, but it also contributes to the development of comorbidities, such as cardiovascular (CV) disease (1, 2).

Immune dysregulation is known to be involved in vascular traits such as endothelial damage, vascular dysfunction, or atherosclerotic plaque development, thus leading to CV disease (3). Although both innate and adaptive immune responses are thought to be involved, the exact mediators are yet to be characterized. Even though a crucial role for T cells and monocytes has been largely hypothesized (4, 5), it has not been until recently that specific, novel subsets of T cells and monocytes were documented to have dedicated functions in vascular homeostasis.

Within the T-cell compartment, angiogenic T cells (Tang) were described as a subset of naive T cells with key roles in vasculogenesis and vascular repair due to their functional cooperation with endothelial progenitor cells (6). As such, Tang have been proven to promote endothelial cell proliferation and function *in vitro* and trigger vessel formation and repair *in vivo* (6). Of note, a decreased frequency of circulating Tang has been reported in several conditions in association with adverse CV outcomes (7–11). However, Tang levels have not been characterized in CKD patients yet. On the other hand, CD4^+^CD28^null^ cells are known to be a biomarker of immunosenescence, and their noxious, cytotoxic-mediated effects on the vasculature have been described *in vitro* and *in vivo*, including endothelial cell apoptosis as well as plaque destabilization and rupture (12–14).

Moreover, recent breakthroughs on monocyte biology need to be considered. First, although classically considered a unique and relative uniform population, monocytes are now recognized as a mixture of phenotypical and functionally different subsets, including at least three categories based on their CD14/CD16 surface expression: classical (CD14^+^CD16^−^), intermediate (CD14^+^CD16^+^), and non-classical (CD14^low^CD16^+^) (15, 16). Since these subsets differ in terms of their ability to elicit cytokine responses, antigen presentation, or phagocytosis (15, 17), it is tempting to speculate that they may have different effects on vascular homeostasis. In addition, changes in the expression of other markers, such as angiotensin-converting enzyme (ACE), have been reported (18, 19) and linked to changes in monocyte activities (19). Even though the role of monocytes in vascular homeostasis has been a topic of major interest, how this monocyte heterogeneity relates to vascular outcomes in CKD remains unknown (20).

Emerging evidence points to an important role of these subsets in vascular homeostasis in other conditions. However, evidence is lacking in CKD. First, most of the studies have focused on individual cell populations, hence making it difficult to compare the effects of the different subsets. Second, vascular involvement in CKD is a complex scenario with multiple stages and pathogenic circuits. Then, focusing on a single entity, such as medial calcification or atherosclerosis does not allow to draw firm conclusions on the global picture. Overcoming these limitations will help not only to gain understanding toward these potential disease mediators but will also pave the ground for their potential use as biomarkers in the clinical setting. Therefore, the main aim of this study was to evaluate potential alterations of different novel immune cell subsets, previously related to vascular homeostasis, as well as their associations with a broad spectrum of vascular surrogate markers, ranging from subclinical atherosclerosis to overt vascular calcification, in CKD patients.

## Materials and Methods

### Ethics Statement

Approval for the study was obtained from the institutional review board (Comité de Ética Regional de Investigación Clínica, reference PI17/02181) in compliance with the Declaration of Helsinki. All participants gave a written informed consent prior to their inclusion in the study.

### Study Participants

Our study involved 33 CKD stage 5 patients undergoing peritoneal dialysis (CKD5-PD) recruited from the Peritoneal Dialysis Outpatient Clinic [Unidad de Gestión Clínica de Nefrología, Hospital Universitario Central de Asturias (HUCA), Spain]. A group of 15 individuals with normal renal function from the general population were recruited as healthy controls (HCs). Additionally, 16 CKD stage 5 patients on hemodialysis (CKD5-HD) (Hemodialysis Outpatient Clinic, HUCA) and six HCs were independently recruited as a replication cohort (Supplementary Table 1). Exclusion criteria were immunosuppressive treatment, pregnancy, diagnosis of immune-mediated disease, cancer or diabetes mellitus, recent (<3 months) or current infections, previous CV disease, abdominal aneurysm or intermittent claudication, or previous carotid surgery. The burden of traditional CV risk factors was assessed by the SCORE algorithm (low-risk charts) according to the European Society of Cardiology (ESC) guidelines (21). The SCORE algorithm integrates information about total cholesterol levels, blood pressure (systolic), smoking status, age, and gender, hence deriving an estimated figure of CV risk (10-year risk for fatal CV). The estimated risk is used in clinical practice to stratify patients [based on ESC guidelines (21)] into categories of risk for preventive pharmacological and non-pharmacological interventions. The SCORE algorithm has been validated in European real-world populations.

Blood samples were obtained by venipuncture (during a clinical appointment before dialysis). Automated serum biochemical parameters, lipid analysis, and complete blood counts were performed on all the participants at the Laboratorio de Medicina (HUCA) by means of routine laboratory methods. Serum samples were stored at −80°C until further analyses.

### Analysis of Peripheral Blood Mononuclear Cells

Peripheral blood samples were immediately processed to obtain peripheral blood mononuclear cells (PBMCs) by centrifugation (1,900 rpm, 20 min) on density gradients (Lymphosep, Biowest, Germany).

The analysis of PBMCs was performed by flow cytometry. First, PBMCs were treated with FcR Blocking Reagent (Miltenyi Biotec, Germany) for 20 min at 4°C to avoid unspecific antibody FcR binding. Then, cells were incubated with CD14 FITC (Immunostep, Spain), CD16 APC-Cy7 (BioLegend, Germany), and ACE APC (Miltenyi Biotec) or CD3 PerCP-Cy-5, 5 (Tonbo Biosciences, Belgium), CD184 PE-Cy7 (BD Biosciences, Germany), CD31 FITC (BD Biosciences), CD4 PE (Immunostep), and CD28 APC-Cy7 (Thermo Fischer, Germany) or corresponding isotype antibodies for 30 min at 4°C protected from light. Next, cells were washed twice with PBS and analyzed by flow cytometry [FACS Canto II (BD Biosciences) with FACS Diva 6.5 software].

**Figure 1:**
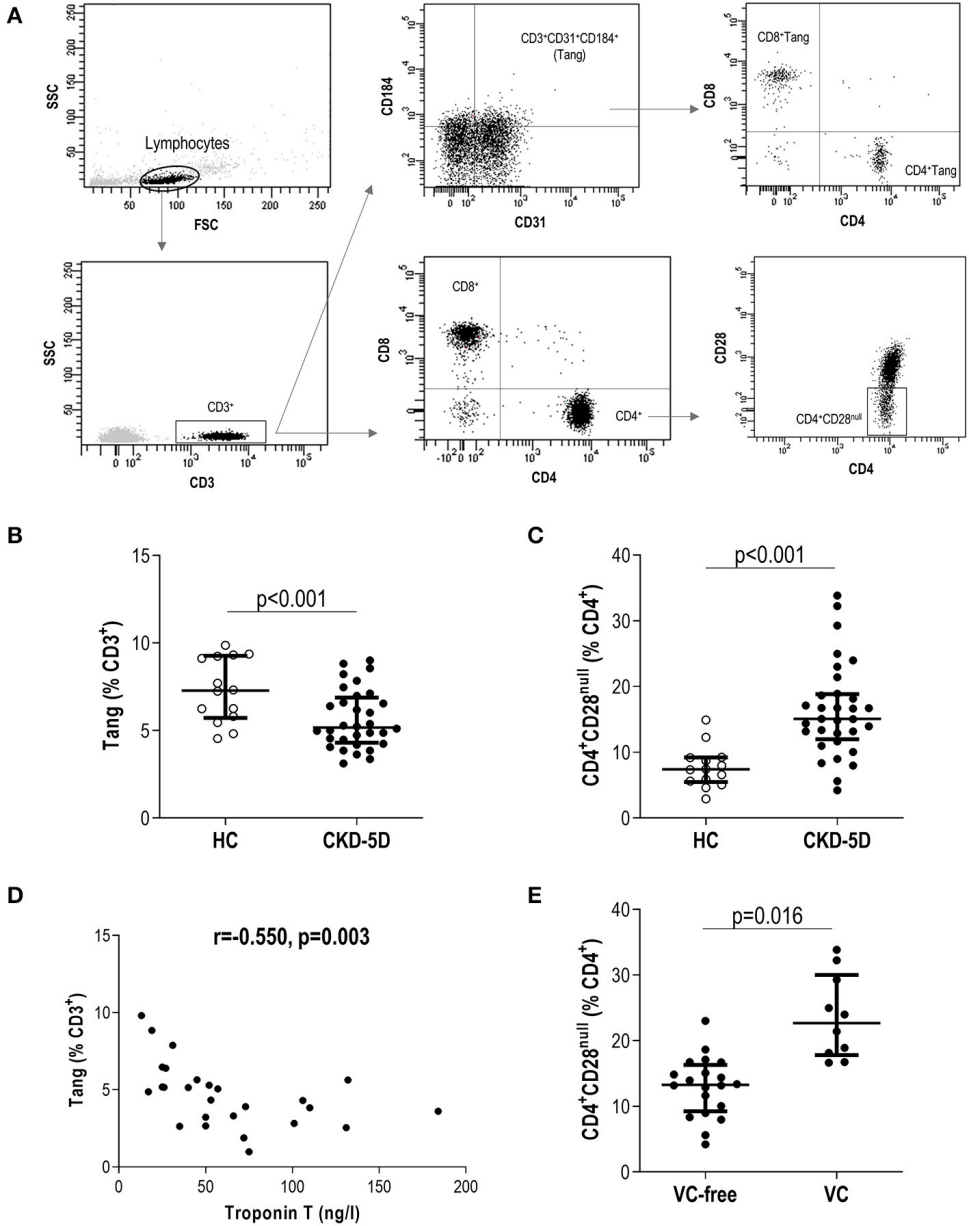
Analysis of novel T-cell subsets in chronic kidney disease (CKD). **(A)** Gating strategy for the identification and quantification of CD3^+^, CD4^+^, CD8^+^, angiogenic T cells (Tang), CD4^+^Tang, CD8^+^Tang, and CD4^+^CD28^null^ subsets by flow cytometry. Dot-plots are shown from a representative patient. Gating was based on isotype controls (Supplementary Figure 1). **(B)** Tang (g = 1.92) and (**C**) CD4^+^CD28^null^ (g = 2.10) levels were compared between healthy control (HC) (open dots) and chronic kidney disease stage 5 patients undergoing peritoneal dialysis (CKD5-PD) (black dots). **(D)** Association between Tang levels and those of serum Troponin T in CKD5-PD. Correlation was assessed by Spearman's rank test. **(E)** Association between CD4^+^CD28^null^ cells and presence of vascular calcification (VC) (g = 1.62). In scatter plots, each dot represents one individual. Upper and lower bars represent 75th and 25th percentiles, and medium bars correspond to the median values. Differences between groups were assessed by Mann–Whitney *U* tests, and size effects were calculated with Hedge's g statistic.

**Figure 2:**
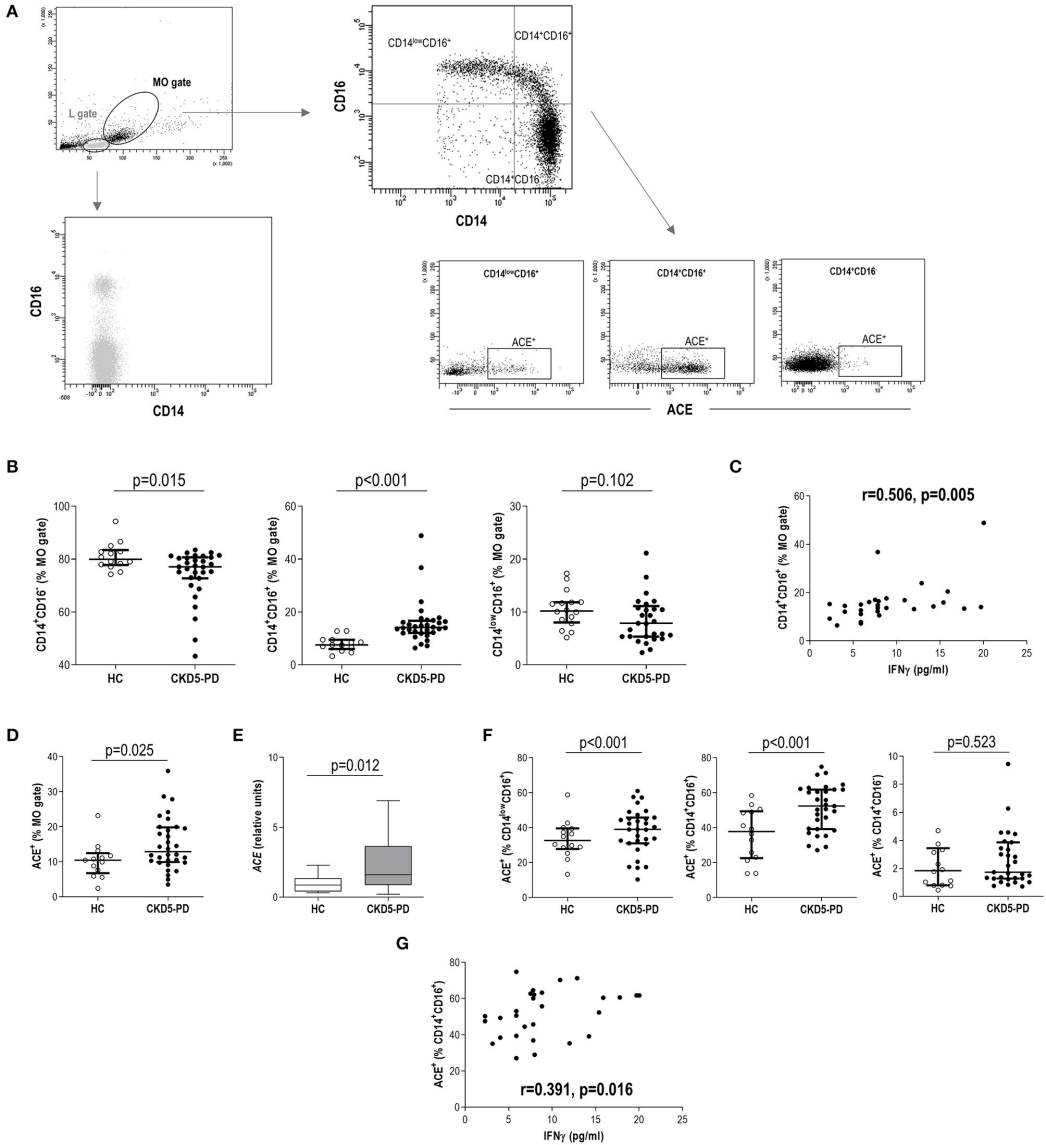
Analysis of monocyte subsets and angiotensin-converting enzyme (ACE) expression in chronic kidney disease (CKD). **(A)** Gating strategy for the identification and quantification of monocyte subsets and their ACE expression. Dot-plots are shown from a representative patient. Monocytes were initially gated from their forward scatter (FSC)/side scatter (SSC) properties (MO gate). Monocytes were not contained within lymphocytes (L gate), which was only composed of CD14^−^ cells. **(B)** Intermediate (g = 0.91) and classical (g = 0.72) monocyte subsets were found to be altered in chronic kidney disease stage 5 patients undergoing peritoneal dialysis (CKD5-PD) (black dots) compared to healthy controls (HCs) (open dots). **(C)** Association between intermediate monocyte levels and those of serum interferon (IFN)γ in CKD5-PD. **(D)** The expression of ACE within the total monocyte gate was analyzed by flow cytometry **(A)**, and increased expression was observed in CKD5-PD (g = 0.90). This was confirmed by gene expression analysis on peripheral blood mononuclear cells (PBMCs) [**(E)**, g = 0.63]. **(F)** The ACE expression was further evaluated on each monocyte subset and compared between HC (open dots) and CKD5-PD (black dots) (non-classical, g = 0.95, and intermediate, g = 1.25). **(G)** Association between ACE^+^ intermediate monocyte and IFNγ serum levels in CKD5-PD. In scatter plots, each dot represents one individual. Upper and lower bars represent 75th and 25th percentiles, and medium bars correspond to the median values. Differences between groups were assessed by Mann–Whitney *U* tests, and size effects were calculated with Hedge's g statistic. Correlations were assessed by Spearman's rank test.

Then, “live gate” excluding debris and no cellular events was designed. Lymphocyte and monocyte regions were defined according to their forward scatter (FSC)/side scatter (SSC) features. Gating was performed based on the signal provided by the isotype controls (Supplementary Figure 1). Tang were defined as previously described (9). In brief, lymphocytes were evaluated for CD3 expression, and those CD31^+^CD184^+^ within the CD3^+^ gate were considered Tang. Tang were further subdivided into CD4^+^Tang and CD8^+^ Tang subsets (Figure 1A). CD3^+^ cells were evaluated for CD4 and CD8 expression, and CD4^+^ cells lacking CD28 expression were defined as CD4^+^CD28^null^ cells (22) (Figure 1A). For the analysis of monocytes, events contained in the monocyte region (FSC/SSC) were evaluated for their CD14 and CD16 expression, and subsets were defined as follows: classical (CD14^+^CD16^−^), intermediate (CD14^+^CD16^+^), and non-classical (CD14^low^CD16^+^) monocytes. All monocytes were contained within the FSC/SSC-defined monocyte region. Moreover, no lymphocytes were found within this region, as these cells were all characterized by a CD14^−^ expression (Figure 2A). No monocytes were observed within the lymphocyte gate either. Finally, ACE expression was evaluated in the whole monocyte region as well as in each of the different monocyte subsets; ACE^+^ events within each population were calculated (Figure 2A). Absolute levels were computed by applying the lymphocyte/monocyte counts obtained in the automated blood cell counts.

### Quantification of Circulating Cytokines

Circulating interleukin (IL)-10, IL-6, IL-2, tumor necrosis factor (TNF)α and interferon (IFN)γ levels were measured in serum samples using a bead-based multiplex assay (BiolegendPlex, BioLegend), following the protocol provided by the manufacturer. Samples were analyzed in a FACS Canto II flow cytometer (BD Biosciences) under FACS Diva 6. The detection limits were 1.2 pg/ml (IL-10 and IL-2) or 2.4 pg/ml (IL-6, TNFα and IFNγ).

### Angiotensin-Converting Enzyme Gene Expression Analysis

The gene expression of ACE was analyzed in total RNA extracted from PBMCs by using TRI reagent (Sigma-Aldrich). After reverse transcription using a High-Capacity cDNA Reverse Transcription Kit (Applied Biosystems), quantitative real-time PCR (qPCR) reactions were performed in triplicate using the Stratagene Mx3005P QPCR System (Agilent Technologies), Fast Start Universal Probe Master (Roche), and pre-developed assays for qPCR [ACE: Hs00174179_m1 and glyceraldehyde 3-phosphate dehydrogenase (GAPDH): Hs99999905_m1; Thermo-Fisher Scientific]. ACE expression was quantified as relative units to GAPDH expression by comparing threshold cycles using the ^ΔΔ^Ct method.

### Surrogate Vascular Outcomes

Abdominal aortic calcification [Kauppila Index (KI)] was quantified in lateral lumbar X-rays. Carotid–femoral pulse wave velocity (PWV) was measured using Complior Analyze (ALAM Medical) according to the manufacturer's instructions. Results are the average of three optimal measurements.

B-mode ultrasound was performed by an experienced radiologist using a Toshiba-Aplio XG machine (Toshiba American Medical Systems). To evaluate left carotid intima-media thickness (cIMT) as well as carotid and femoral plaque presence (either cIMT >1.5 mm or a focal thickening going over into the arterial lumen by at least 50% of the surrounding cIMT value) (23), all subjects, laid in a supine position, underwent a prior axial examination of the extracranial carotid artery followed by a longitudinal exploration.

Plaques were described by number, location, dimension, echogenicity, and homogeneity (stable and unstable).

Adventitial vasa vasorum (aVV), the plexus of microvessels surrounding the adventitial layer, were quantified following a similar strategy to that validated by Arcidiacono et al. (24) but avoiding the use of a contrast agent. Indeed, the very sensitive Superb Microvascular Imaging Ultrasound (Toshiba Aplio 500), designed for the detection of small-diameter blood vessel flow, was used. Vasa vasorum density was quantified in the left carotid artery and, for the first time, in the femoral arteries of HC and CKD5-PD (Supplementary Figure 2). The ImageJ program was used for the quantification of aVV density of the adventitial vessels, identified by an experienced radiologist, as depicted in representative Supplementary Figure 2. All surrogate vascular measurements were performed by an operator blinded to the study participants.

### Statistical Analyses

Continuous variables were expressed as median [interquartile range (IQR)] or mean ± standard deviation according to the distribution of the variables. Categorical variables were summarized as *n* (%). Differences between groups were assessed by Mann–Whitney *U* tests or Student *T* tests, as appropriate. Hedge's g statistic was calculated in order to evaluate size effects (values g > 0.6 and g > 0.80 were considered of medium effect and large effect, respectively) (25). The associations between continuous variables were evaluated by correlations (Spearman's rank test) or linear regression models, either univariate or multivariate adjusted by confounders. B coefficients and 95% confidence intervals (CIs) were computed. A *p*-value < 0.050 was considered statistically significant. Statistical analyses were performed with SPSS 24.0 and GraphPad Prism 8.0 for Windows.

## Results

### Novel T-Cell Subsets in Chronic Kidney Disease

A total of 33 CKD5-PD and 15 HCs were recruited for this study (Table 1). Tang (CD3^+^CD31^+^CD184^+^) levels in peripheral blood were evaluated by flow cytometry, and a highly significant lower Tang frequency was observed in CKD5-PD (Figure 1B). No differences were found in the frequency of CD4^+^ [46.55% (21.07) vs. 48.66% (19.45), *p* = 0.518] and CD8^+^ subsets [42.20% (21.01) vs. 41.09% (18.16), *p* = 0.258] within the Tang pool between patients and controls. As a consequence, both CD4^+^Tang [1.88% (1.16) vs. 3.43% (1.80), *p* < 0.001, g = 1.82] and CD8^+^Tang [2.02% (0.88) vs. 3.11% (1.72), *p* < 0.001, g = 1.15] were found to be decreased in CKD5-PD. Moreover, CD4^+^CD28^null^ cells were also evaluated, and CKD5-PD exhibited a marked increase in this cell population compared to HC (Figure 1C). These differences were maintained when evaluated within the total lymphocyte region [Tang: 1.68% (1.05) vs. 3.38% (1.44), *p* < 0.001, g = 0.92; and CD4^+^CD28^null^: 9.06% (4.16) vs. 5.09% (2.52), *p* < 0.001, g = 1.10]. Importantly, equivalent results were observed when the absolute levels were computed [Tang: 2.38 (2.29) vs. 7.00 (4.99)·10^3^/μl, *p* < 0.001, g = 2.30; CD4^+^CD28^null^: 11.65 (8.83) vs. 5.82 (10.34)·10^3^/μl, *p* = 0.035, g = 0.70]. Similarly, no differences were observed in the CD3^+^ [HC: 46.25% (17.39) vs. CKD: 42.26% (29.55), *p* = 0.447), CD4^+^ (61.86% (12.55) vs. 60.99% (14.81), *p* = 0.645] or CD8^+^ subsets [33.86% (12.95) vs. 34.85% (12.00), *p* = 0.800].

**Table 1:** Demographical, laboratory, and clinical parameters of individuals recruited for this study.

	**HC (*n* = 15)**	**CKD5-PD (*n* = 33)**	***P*-value**
Age, years, mean (range)	48.00 (22.00–68.00)	55.00 (21.00–77.00)	0.070
Sex, women/men	10/5	13/20	0.080
**Laboratory parameters**			
Albumin, mg/dl	44.17 ± 1.95	34.37 ± 4.35	< 0.001
Urea, mg/dl	33.46 ± 8.08	132.66 ± 43.76	< 0.001
Creatinine, mg/dl	0.78 ± 0.17	7.81 ± 2.72	< 0.001
Plasma Ca, mmol/l	2.34 ± 0.07	2.17 ± 0.16	< 0.001
Plasma phosphate, mmol/l	1.04 ± 0.18	1.64 ± 0.42	< 0.001
PTH, pg/ml	49.60 ± 15.52	371.03 ± 198.61	< 0.001
Total cholesterol, mg/dl	202.25 ± 49.20	159.32 ± 40.28	0.005
HDL cholesterol, mg/dl	67.63 ± 13.14	59.00 ± 24.52	0.017
LDL cholesterol, mg/dl	113.00 ± 38.38	74.71 ± 38.56	0.002
Triglycerides, mg/dl	65.53 ± 25.54	131.82 ± 75.33	0.003
25(OH)-vitamin D, ng/ml	32.93 ± 15.38	10.10 ± 6.89	< 0.001
CRP, mg/dl	0.14 ± 0.07	0.64 ± 0.86	0.002
Troponin T, ng/l	5.33 ± 3.45	61.48 ± 41.82	< 0.001
**Blood cell counts (10**^**3**^**/μl) median (IQR)**			
Leukocytes	5.67 (2.06)	6.25 (3.59)	0.380
Neutrophils	2.94 (1.15)	3.52 (2.27)	0.075
Lymphocytes	1.96 (0.91)	1.22 (0.85)	0.022
Monocytes	0.44 (0.19)	0.56 (0.23)	0.071
Eosinophils	0.11 (0.10)	0.23 (0.20)	0.002
Basophils	0.04 (0.02)	0.05 (0.05)	0.109
**Clinical features**			
Vascular calcifications, n (%)		18 (54.5)	
Kauppila score		7.88 ± 8.98	
Time on dialysis, months (median (IQR)		15.00 (16.00)	
Systolic blood pressure, mm Hg		133.88 ± 16.04	
Diastolic blood pressure, mm Hg		81.05 ± 10.70	
**Treatments, n (%)**			
Paricalcitol		15 (45.4)	
Phosphate binders		18 (54.5)	
Statins		21 (63.6)	
Methylprednisolone^‡^		9 (27.22)	
Epo		24 (72.7)	

*Variables were summarized as mean ± SD or n (%), unless otherwise stated. Differences were assessed by Student t tests, Mann–Whitney U tests, or χ^2^ tests, as appropriate. ^‡^ 8/9 patients were under low-dose glucocorticoids (<5 mg/day)*.

*CKD5-PD, chronic kidney disease stage 5 patients undergoing peritoneal dialysis; CRP, C-reactive protein; HC, healthy control; HDL cholesterol, high-density lipoprotein cholesterol; IQR, interquartile range; LDL cholesterol, low-density lipoprotein cholesterol*.

Next, in order to gain insights into the origin of these alterations, their associations with laboratory parameters and clinical features were analyzed. Whereas, Tang levels were associated with those of total cholesterol (*r* = −0.665, *p* = 0.005) and low-density lipoprotein (LDL) cholesterol (*r* = −0.372, *p* = 0.021) in HC, these associations were not found in patients (*r* = −0.037, *p* = 0.853 and *r* = −0.203, *p* = 0.301, respectively). Interestingly, Tang levels showed an inverse correlation with serum troponin T (TnT) concentration in CKD5-PD (Figure 1D). Regarding CD4^+^CD28^null^ cells, patients with vascular calcifications exhibited higher levels of this subset than their calcification-free counterparts (Figure 1E). No associations were observed for Tang or CD4^+^CD28^null^ cells with the rest of biochemical parameters examined in either HCs or patients [all *p* > 0.050, or with time on dialysis or medications in patients (all *p* > 0.050)]. Glucocorticoid usage was not observed to have an effect on these subpopulations (Tang: *p* = 0.356, CD4^+^CD28^null^: *p* = 0.239). Excluding patients under glucocorticoid treatment yielded equivalent results. Similarly, no associations were observed between Tang or CD4^+^CD28^null^ cells with the levels of circulating cytokines (Supplementary Tables 1, 2).

Finally, we recruited a replication cohort consisting of 16 CKD5-HD and six age- and gender-matched controls (Supplementary Tables 3, 4) in order to validate our results. The independent analysis of this cohort confirmed the differences observed in peritoneal dialysis patients for these cell subsets (Supplementary Figure 2). No major differences in blood cell counts and clinical parameters were observed between CKD5-HD and their peritoneal dialysis counterparts.

These results suggest that CKD is hallmarked by distinct alterations of T-cell subsets.

### Monocyte Subsets and Angiotensin-Converting Enzyme Expression

The analysis of monocyte subsets (Figure 2A) revealed an increased frequency of intermediate monocytes in CKD5-PD at the expense of a reduction in the classical group compared to HC (Figure 2B). Equivalent results were observed when absolute levels were calculated [intermediate: 7.67 (5.61) vs. 3.89 (3.71)·10^3^/μl, *p* < 0.001, g = 0.89; classical: 31.22 (17.88) vs. 42.18 (18.35)·10^3^/μl, *p* = 0.035, g = 0.62; non-classical: 3.90 (4.10) vs. 4.10 (2.28)·10^3^/μl, *p* = 0.696]. Intermediate monocytes were found to be positively correlated with IFNγ serum levels (Figure 2C), whereas no other associations were observed with the rest of cytokines analyzed. No effect was observed for medications (all *p* > 0.050), including glucocorticoids (intermediate: *p* = 0.128, classical: *p* = 0.318, non-classical: *p* = 0.564). Excluding patients under glucocorticoid treatment yielded equivalent results.

Additionally, the surface expression of ACE was analyzed in monocytes. Monocytes from CKD5-PD exhibited an increased ACE expression (Figure 2D). This increase was confirmed at the gene expression level by RT-PCR (Figure 2E). Interestingly, the ACE expression was not uniform within the monocyte pool, but differences were noted among subsets. It was mainly expressed by intermediate and non-classical monocytes, whereas their classical counterparts exhibited a negligible expression (Figure 2A). Moreover, increased expression was observed in CKD5-PD compared to controls in both intermediate and non-classical subsets compared to HC (Figure 2F). Of note, ACE expression (analyzed by flow cytometry) on intermediate monocytes was found to be positively correlated with IFNγ serum levels (Figure 2G).

Furthermore, equivalent analyses were performed in our replication cohort, and similar results were obtained (Supplementary Figure 3).

All these findings suggest that quantitative and qualitative differences characterize the monocyte pool in CKD patients, linked at least in part to IFNγ levels.

### Novel Immune Cell Subsets and Vascular Surrogate Markers

Next, vascular surrogate markers (including aVV, PWV, cIMT) and Kauppila scores were measured in 17 CKD5-PD and 14 HCs. No differences in demographical, clinical, medications, or blood counts were observed between patients with and without vascular assessments, thus indicating that the subset analyzed was representative of the whole population (Supplementary Table 5). Of note, patients exhibited higher carotid aVV expansion and a trend in femoral location, together with higher PWV and cIMT values than HCs (Table 2).

**Table 2:** Vascular surrogate markers in CKD and HC.

	**HC (*n* = 14)**	**CKD5-PD (*n* = 17)**	***P*-values**
Carotid aVV, number (n vasa)	0.00 (2.00)	3.00 (7.00)	< 0.050
Carotid aVV, area (% mm^2^/mm)	0.00 (2.16)	2.61 (8.84)	0.023
Femoral aVV, number (n vasa)	0.00 (1.25)	1.00 (3.50)	0.245
Femoral aVV, area (% mm^2^/mm)	0.00 (1.57)	0.89 (4.67)	0.121
PWV, m/s	7.20 (1.38)	8.70 (2.82)	0.008
cIMT, mm	0.60 (0.20)	0.75 (0.32)	0.045

*Variables were summarized as median (interquartile range) and compared by Mann–Whitney U tests*.

*aVV, adventitial vasa vasorum; cIMT, carotid intima-media thickness; CKD5-PD, chronic kidney disease stage 5 patients undergoing peritoneal dialysis; HC, healthy control; PWV, pulse wave velocity*.

In order to evaluate whether immune cell populations could be regarded as biomarkers of subclinical CV endpoints, correlations with these assessments were performed (Table 3). Interestingly, Tang were strongly correlated with aVV and PWV measurements. Importantly, these associations were maintained after correcting for the effect of traditional CV risk factors (Table 4). On the other hand, CD4^+^CD28^null^ cells were found to be associated with cIMT and Kauppila score (Table 3). This association was maintained [β (95% CI), *p* = 0.883 (0.357, 1.409), *p* = 0.003] in a multivariate model after adjusting for traditional CV risk factors (entered as the SCORE) and time on dialysis as confounders. Finally, intermediate monocytes were correlated to PWV and vascular calcification (Table 3), although correlations were observed to be weaker than those of T-cell subsets. These associations were not observed after controlling for confounders (both *p* > 0.050). Of note, no associations with other T-cell subsets (CD3^+^, CD4^+^, or CD8^+^) or leukocyte subsets were observed (Supplementary Table 6), thus ruling out a potential confounding effect and emphasizing the specific role of the previous cell populations in vascular repair.

**Table 3:** Associations between subclinical CV outcomes and immune cell subsets in CKD5-PD.

	**Number of neovasa (carotid)**	**Number of neovasa (femoral)**	**aVV area (carotid)**	**aVV area (femoral)**	**PWV**	**cIMT**	**Kauppila**
**T cells**							
Tang (% lymphocytes)	***r*****=−0.640** ***p*****=0.006**	*r* = −0.194 *p* = 0.456	***r*****=−0.813** ***p*****<0.001**	***r*****=−0.542** ***p*****=0.025**	***r*****=−0.734** ***p*****<0.001**	*r* = −0.029 *p* = 0.921	*r* = −0.359 *p* = 0.060
CD4^+^CD28^null^ (% lymphocytes)	*r* = 0.333 *p* = 0.192	*r* = 0.088 *p* = 0.737	*r* = 0.395 *p* = 0.117	*r* = 0.395 *p* = 0.117	*r* = 0.435 *p* = 0.093	***r*****=0.566** ***p*****=0.035**	***r*****=0.581** ***p*****<0.001**
**Monocytes**							
Non-classical (% monocytes)	*r* = 0.286 *p* = 0.265	*r* = −0.325 *p* = 0.203	*r* = 0.505 *p* = 0.039	*r* = 0.069 *p* = 0.794	*r* = 0.271 *p* = 0.310	*r* = −0.237 *p* = 0.415	*r* = 0.217 *p* = 0.258
ACE^+^ Non-classical (% non-classical)	*r* = −0.123 *p* = 0.637	*r* = −0.085 *p* = 0.744	*r* = −0.114 *p* = 0.662	*r* = −0.040 *p* = 0.879	*r* = 0.055 *p* = 0.841	*r* = 0.059 *p* = 0.842	*r* = −0.214 *p* = 0.266
Intermediate (% monocytes)	*r* = 0.294 *p* = 0.252	*r* = 0.167 *p* = 0.522	*r* = 0.472 *p* = 0.086	*r* = 0.378 *p* = 0.135	***r*****=0.576** ***p*****=0.020**	*r* = 0.171 *p* = 0.558	***r*****=0.388** ***p*****=0.037**
ACE^+^ intermediate (% intermediate)	*r* = 0.158 *p* = 0.545	*r* = −0.166 *p* = 0.106	*r* = 0.406 *p* = 0.106	*r* = 0.159 *p* = 0.542	*r* = 0.175 *p* = 0.516	*r* = −0.183 *p* = 0.532	*r* = 0.017 *p* = 0.930
Classical (% monocytes)	*r* = −0.299 *p* = 0.243	*r* = 0.065 *p* = 0.804	***r*****=−0.559** ***p*****=0.043**	*r* = −0.345 *p* = 0.174	*r* = −0.473 *p* = 0.064	*r* = 0.088 *p* = 0.765	*r* = −0.279 *p* = 0.142
ACE^+^ classical (% classical)	*r* = 0.257 *p* = 0.319	*r* = −0.013 *p* = 0.961	*r* = 0.262 *p* = 0.310	*r* = 0.148 *p* = 0.572	*r* = 0.251 *p* = 0.349	*r* = −0.083 *p* = 0.777	*r* = 0.326 *p* = 0.090

*Correlations were assessed by Spearman's rank tests. Correlations reaching statistical significance were highlighted in bold*.

*ACE, angiotensin-converting enzyme; aVV, adventitial vasa vasorum; cIMT, carotid intima-media thickness; CKD5-PD, chronic kidney disease stage 5 patients undergoing peritoneal dialysis; CV, cardiovascular; PWV, pulse wave velocity; Tang, angiogenic T cells*.

**Table 4:** Tang as predictors of subclinical CV outcomes.

	**Number of neovasa (carotid)**	**Number of neovasa (femoral)**	**aVV area** **(carotid)**	**aVV area** **(femoral)**	**PWV**
**Univariate**					
Tang	−0.505 (−2.281, −0.485) *p* = 0.004	−0.293 (−1.085, 0.116) *p* = 0.110	−0.523 (−3.819, −0.862) *p* = 0.003	−0.466 (−2.936, −0.477) *p* = 0.008	−0.663 (−1.40, −0.682) *p* < 0.001
**Multivariate**					
Tang	−0.662 (−6.309, −0.582) *p* = 0.022	0.161 (−1.290, 2.263) *p* = 0.566	−2.313 (−10.870, −0.409) *p* = 0.036	−0.393 (−6.963, −0.010) *p* = 0.048	−0.437 (−3.390, −0.141) *p* = 0.010
SCORE	−0.270 (−2.055, 0.702) *p* = 0.310	0.540 (−0.071, 1.640) *p* = 0.069	0.025 (−2.404, 2.633) *p* = 0.924	0.411 (−0.258, 3.423) *p* = 0.086	0.368 (−0.241, 1.545) *p* = 0.139

*The associations between Tang frequency and subclinical CV outcomes were analyzed by linear regression in univariate or multivariate models adjusted for the burden of traditional CV risk factors (entered as the SCORE). The unstandardized regression coefficient (B) and (95% CI) with the corresponding p-values were computed*

*aVV, adventitial vasa vasorum; CV, cardiovascular; PWV, pulse wave velocity; Tang, angiogenic T cells*

*The associations between Tang frequency and subclinical CV outcomes were analyzed by linear regression in univariate or multivariate models adjusted for the burden of traditional CV risk factors (entered as the SCORE). The unstandardized regression coefficient (B) and (95% CI) with the corresponding p-values were computed*

*aVV, adventitial vasa vasorum; CV, cardiovascular; PWV, pulse wave velocity; Tang, angiogenic T cells*

Taken together, distinct altered immune cell populations are related to different vascular surrogate outcomes in CKD. Tang were independently associated with subclinical atherosclerosis and vascular functionality even after adjusting for traditional risk factors.

## Discussion

Although systemic inflammation has been described to contribute to vascular outcomes in CKD, the exact mediators are yet to be characterized. A growing body of evidence has identified novel immune cell subsets that play a role in vascular homeostasis and damage in different scenarios. CKD patients suffer a profound vascular deterioration, with different mechanisms being involved. Delineating the associations of the different immune mediators with the distinct adverse vascular scenarios is of major interest. In the present study, we addressed the analysis of some of these immune cell subsets in CKD. Our findings revealed marked alterations within T cells subsets, hallmarked by a Tang decrease and elevated CD4^+^CD28^null^ cells, as well as within the monocyte pool, characterized by a shift toward CD16^+^ subsets and enhanced ACE expression. These alterations exhibited distinct associations with vascular outcomes in patients under peritoneal dialysis, Tang depletion being related to a poor vascular functionality and subclinical atherosclerosis, whereas senescent CD4^+^ T cells were associated with overt vascular wall thickening and calcification. Of note, these alterations were found in CKD5 patients undergoing peritoneal or hemodialysis compared to their control populations, thus suggesting that these phenomena may be related to the CKD stage itself. To the best of our knowledge, this is the first study not only to present a joint characterization of these subsets in CKD but also to evaluate their associations with a broad range of surrogate vascular outcomes in this condition.

One of the most remarkable findings of our work was the analysis of the Tang population. Tang are known to carry out vascular protective actions (6), and Tang depletion has been linked to CV outcomes in several immune-mediated conditions (7–11). The results herein presented expand the current knowledge in a two-fold manner. First, Tang depletion was observed beyond autoimmunity, hence strengthening the role of Tang as potential mediators of vascular homeostasis in a broad range of disorders, rather than being an immune disease-specific mechanism. Importantly, both CD4^+^ and CD8^+^ Tang subsets were found to be diminished, pointing to a strong quantitative effect on the Tang subset rather than a qualitative effect within the Tang composition. Second, decreased Tang have been linked to subclinical CV disease surrogate markers (aVV and PWV), that is, with the first signs of vascular impairments, but not with more advanced vascular traits (such as vascular calcification). In fact, in healthy individuals with zero risk for the classical atherosclerotic risk factors, left carotid aVV increased with age, the natural determinant of a higher prevalence of atheromatous lesions, in parallel with increases in cIMT within the normal range, thus supporting the accuracy and sensitivity of aVV as an earlier marker of subclinical atherosclerosis (24). Furthermore, in experimental hypercholesterolemia, increases in the density of coronary aVV precede epicardial endothelial dysfunction (26). Accordingly, type 2 diabetic patients present a higher left carotid adventitial neovascularization when compared to controls, with the highest aVV density in patients with retinopathy (angiogenesis) despite similar cIMT (27). Also, increases in aVV and in cIMT also occur in the right carotid artery of CKD stages 3–4 and dialysis patients with higher neovascularization at the earlier CKD stages (28).

Similarly, PWV is a well-established early marker of arterial stiffness (29). Since CKD patients exhibited both atherosclerosis and arteriosclerosis (30, 31), finding a mediator linked to the subclinical stage of both processes is of major relevance. Furthermore, this is supported by the strong association with TnT levels in CKD, a well-known biomarker of subclinical CV damage with predictive ability in CKD (32, 33). Therefore, a role for Tang to stratify CV risk may be considered. Interestingly, aVV expansion has been reported to be dependent on hypoxia-inducible factor (HIF)-1 and vascular endothelial growth factor (VEGF) production (34, 35), these molecules being also linked to Tang mobilization (6). Importantly, current literature points to an association between aVV sprouting and T-cell adventitial accumulation, rather than monocytes or other subsets (36–38). These notions are in line with our findings comparing the distinct vascular surrogate markers. Taken together, these results may point to Tang as potential biomarkers of subclinical vascular impairment in CKD.

Furthermore, Tang depletion was not associated with the burden of traditional CV risk factors, and they were confirmed to be independent predictors of vascular surrogate markers. Therefore, this reinforces the idea that Tang could provide additional information as biomarkers. This is further supported by the fact that Tang depletion seemed to be a uniform observation in CKD patients, that is, linked to the disease itself and not to specific disease features. Since current algorithms solely based on traditional risk factors fail to achieve an appropriate risk stratification in CKD (39–41), these findings may support the use of Tang as an additional instrument in the clinical setting. Larger studies to explore the ability of Tang to reclassify CKD patients to appropriate risk categories are warranted.

Additionally, the analysis of T-cell subsets revealed that CKD patients were hallmarked by a CD4^+^CD28^null^ expansion. This subset was strongly correlated with the extent of vascular calcification, whereas no associations with subclinical features were noted. Interestingly, CD4^+^CD28^null^ are terminally differentiated cells that result from a cumulative T-cell activation, leading to T cells exhaustion (42, 43). As a consequence, they are originated after a long exposure to pro-senescent stimuli. This notion may underlie their association with vascular calcification, as it is the result of an evolutive, cumulative process, thus supporting their association with a harder endpoint than subclinical vascular outcomes, as observed for Tang.

Taken together, these lines of evidence highlight that T cells may act as a double-edged sword for vascular outcomes in inflammatory conditions. Despite being highly heterogeneous, T cells have been largely considered to prompt vascular impair or endothelial damage progression. The observation that early T-cell accumulation precedes vascular lesions further supported this idea. However, the findings from our group (8, 9) and others (7, 10, 44) support the existence of T cell-mediated protective mechanisms for vascular homeostasis. Disruptions of these mechanisms or an altered balance with the deleterious effects mediated by other T-cell populations may explain the occurrence of vascular events (45). Then, the role of T cells in this scenario may be more complex than initially conceived. Consequently, the protective effects of some T-cell subsets may be taken into account, especially when the use of immunomodulating agents is considered.

Of note, despite our results showing profound changes in monocyte subsets in CKD patients, only intermediate monocytes were related to vascular surrogate markers, such correlations being weaker than those retrieved for T cells and being interfered by potential confounders. These results may sound counterintuitive, since monocytes are pivotal for the development of some vascular events. Moreover, the lack of association with clinical features leads us to think that monocyte alterations may be related to the immunopathogenesis of the CKD itself, rather than to vascular outcomes. Interestingly, the notion that a Th1 predominance has been documented in CKD (46, 47) aligns with this point. The fact that the disturbances of the circulating monocytes seemed to be homogeneous across all patients independently of clinical features (even between dialysis modalities) prompted us to hypothesize that these alterations may appear earlier in the continuum of CKD stages, thus being a common trait of its pathogenesis. This may explain why these changes were so consistent across individuals. Importantly, animal models have also confirmed an early shift in monocyte subsets during the initial steps leading to CKD (48). Therefore, it may be conceivable that monocytes become activated in previous stages, thus eliciting an inflammatory response by activating adaptive responses at a later stage. Emerging evidence supports this “progressive” model, with an initial involvement of innate pathways that fuel an aberrant activation of adaptive responses in the final phases of CKD. These lines of evidence may account for the lack of associations between monocyte disturbances and vascular outcomes in CKD stage 5.

In conclusion, novel immune cell subsets are related to vascular surrogate markers in CKD patients. T cells emerge as double-edged swords in the associations between vascular outcomes and CKD, with Tang depletion being associated with poor vascular functionality and subclinical atherosclerosis, whereas CD4^+^CD28^null^ were related to calcification. On the contrary, profound quantitative and qualitative changes were found within the monocyte compartment, but only intermediate monocytes were slightly related to vascular outcomes in univariate analyses. Our proof-of-concept study suggested for the first time a potential role for Tang in CKD, hence paving the ground for functional studies to unveil the mechanisms underlying Tang depletion in CKD. However, this study comes with some limitations that should be remarked, including reduced sample size and cross-sectional design. Our patient population was homogeneous and reflected a real-world CKD population undergoing dialysis. However, patients with diabetes were excluded due to the autoimmune background, which might have a differential effect on these populations, independently of that of the CKD. Further studies with appropriate control populations are needed to evaluate whether the alterations herein reported could be observed in diabetic CKD patients. The inclusion of an independent validation cohort strengthens the consistency of our findings. It must be noted that our study found validation in the independent comparisons of each CKD5 cohort with its respective control group, but no comparisons were made between dialysis modalities, as this felt beyond the study aims. Having found a similar picture in these analysis, our findings could be considered to be a general effect of the CKD stage itself, although a potential effect of the dialysis modality should not be ruled out. However, whether these alterations may be extended to other CKD stages needs to be confirmed in future studies. Similarly, whether dialysis modality may influence these alterations cannot be addressed in the present study. Importantly, the findings related to the vascular outcomes were only analyzed in patients undergoing PD, so it remains unclear whether these results can be transferred to patients under hemodialysis. Furthermore, larger, long-term prospective studies are warranted to evaluate whether these subsets could be considered predictive biomarkers that could assist in the clinical setting.

## Data Availability Statement

The original contributions presented in the study are included in the article/Supplementary Material, further inquiries can be directed to the corresponding author/s.

## Ethics Statement

The studies involving human participants were reviewed and approved by Comité de Ética Regional de Investigación Clínica. The patients/participants provided their written informed consent to participate in this study.

## Author Contributions

JR-C and NC-L performed most of the experimental procedures and carried out the statistical analyses. CU and BM-C carried out some experimental procedures. CR-S, ES-A, and MR-G were in charge of patients' recruitment and clinical data collection. JR-C, NC-L, MN-D, MA, BF-M, JC-A, AS, and AD contributed to the data analysis interpretation and discussion of the results. JR-C, NC-L, and AS drafted the manuscript. AD conceived the study, designed the protocols, and edited the manuscript. All authors read the manuscript, revised it for intellectual content, approved the final version, and agreed to be accountable for all aspects of the work.

## Conflict of Interest

The authors declare that the research was conducted in the absence of any commercial or financial relationships that could be construed as a potential conflict of interest.
