# Supplementary material for: Novel Immune Cell Subsets Exhibit Different Associations With Vascular Outcomes in Chronic Kidney Disease Patients—Identifying Potential Biomarkers
Source: Front Med (Lausanne). 2021 May 25;8:618286. doi: 10.3389/fmed.2021.618286 (PMC8185045; doi:10.3389/fmed.2021.618286)

**SUPPLEMENTARY MATERIALS**

**Supplementary Table 1: Serum levels of circulating cytokines in controls and CKD5-PD patients.** Cytokine levels were summarized as median (interquartile range) and compared by Mann-Withney U tests.

| ***Cytokines, pg/ml*** | **HC**  **(n=15)** | **CKD5-PD**  **(n=33)** | **p-value** |
| --- | --- | --- | --- |
| IL-2 | 1.30 (1.91) | 1.60 (0.70) | 0.342 |
| IL-6 | 4.73 (4.58) | 6.77 (10.07) | 0.154 |
| IL-10 | 1.49 (0.80) | 1.77 (1.05) | 0.101 |
| IFNγ | 8.32 (6.80) | 12.82 (6.57) | 0.183 |
| TNFα | 8.38 (7.66) | 14.01 (6.38) | 0.211 |

**Supplementary Table 2: Analyses of correlations between circulating immune T-cell subsets (Tang and CD4+CD28null) and serum levels of cytokines in CKD5-PD.** Correlations were assessed by Spearman ranks’ tests.

|  | **Tangcells** | **CD4+CD28null** |
| --- | --- | --- |
| IL-2 | r=0.188, p=0.328 | r=0.176, p=0.361 |
| IL-6 | r=0.231, p=0.228 | r=0.283, p=0.137 |
| IL-10 | r=0.251, p=0.190 | r=0.180, p=0.349 |
| IFNg | r=0.178, p=0.355 | r=0.170, p=0.378 |
| TNFa | r=0.189, p=0.325 | r=0.271, p=0.150 |

**Supplementary Table 3: Demographical, clinical and immunological characteristics of individuals from the replication cohort.** Variables were summarized as mean±SD or n(%), unless otherwise stated. Differences were assessed by Student t tests, Mann-Withney U tests or χ2 tests, as appropriate.

|  | **HC**  **(n=6)** | **CKD5-HD**  **(n=16)** | **p-value** |
| --- | --- | --- | --- |
| Age, years, mean (range) | 57.00 (49.00 – 67.00) | 54.87 (21.00 – 74.00) | 0.589 |
| Sex, women/men | 2/4 | 6/10 | 0.856 |
|  |  |  |  |
| ***Clinical features*** |  |  |  |
| Albumin, mg/dl | 44.08±2.07 | 36.08±6.78 | <0.001 |
| Urea, mg/dl | 38.16±7.08 | 100.93±29.95 | <0.001 |
| Creatinine, mg/dl | 0.99±0.20 | 8.19±2.26 | <0.001 |
| Plasma Ca, mmol/l | 2.47±0.08 | 2.19±0.22 | 0.032 |
| Plasma phosphate, mmol/l | 1.01±0.13 | 1.49±0.60 | 0.015 |
| PTH, pg/ml | 50.20±15.67 | 379.31±321.74 | <0.001 |
| Total cholesterol, mg/dl | 235.16±38.65 | 152.56±52.59 | 0.002 |
| HDL-cholesterol, mg/dl | 74.33±13.20 | 46.81±14.57 | 0.002 |
| LDL-cholesterol, mg/dl | 131.00±29.19 | 73.50±46.93 | 0.032 |
| Triglycerides, mg/dl | 103.33±42.05 | 167.75±100.25 | 0.052 |
| 25(OH)-vitamin D, ng/ml | 29.72±8.59 | 8.32±5.85 | <0.001 |
| CRP, mg/dl | 0.24±0.09 | 0.38±0.36 | 0.002 |
| Troponin T, ng/l | 4.13±3.28 | 47.12±27.50 | <0.001 |
|  |  |  |  |
| ***Bloodcellcounts (·10^3^/μl)*** |  |  |  |
| Leukocytes | 5.69 (1.71) | 5.98 (2.24) | 0.914 |
| Neutrophils | 3.19 (1.03) | 3.83 (1.85) | 0.367 |
| Lymphocytes | 1.80 (0.80) | 1.38 (0.56) | 0.049 |
| Monocytes | 0.48 (0.39) | 0.64 (0.35) | 0.802 |
| Eosinophils | 0.18 (0.18) | 0.15 (0.13) | 0.641 |
| Basophils | 0.04 (0.03) | 0.03 (0.02) | 0.294 |
|  |  |  |  |
| ***Clinical features*** |  |  |  |
| Vascular calcifications, n(%) |  | 10 (62.5) |  |
| Kauppila score |  | 6.33±4.98 |  |
| Time on dialysis, months |  | 31.00 (54.50) |  |
| Systolic blood pressure, mm Hg |  | 129.06±22.89 |  |
| Diastolic blood pressure, mm Hg |  | 64.00±22.25 |  |
|  |  |  |  |
| ***Treatments, n(%)*** |  |  |  |
| Paricalcitol |  | 7 (43.7) |  |
| Phosphatebinders |  | 11 (68.7) |  |
| Statins |  | 9 (56.2) |  |
| Metilprednisone |  | 4 (25.0) |  |
| Epo |  | 13 (81.2) |  |

**Supplementary Table 4: Serum levels of circulating cytokines in controls and CKD5-HD patients.** Cytokine levels were summarized as median (interquartile range) and compared by Mann-Withney U tests.

| ***Cytokines, pg/ml*** | **HC**  **(n=6)** | **CKD5-HD**  **(n=16)** | **p-value** |
| --- | --- | --- | --- |
| IL-2 | 1.35 (1.05) | 1.62 (0.59) | 0.876 |
| IL-6 | 5.12 (3.75) | 5.10 (3.04) | 0.978 |
| IL-10 | 1.72 (1.07) | 1.50 (1.29) | 0.568 |
| IFNγ | 8.19 (7.10) | 13.14 (8.56) | 0.123 |
| TNFα | 7.79 (5.35) | 10.11 (4.28) | 0.453 |

**Supplementary Table 5: Comparative analysis of patients with and without subclinical CV outcomes.** Variables were summarized as mean±SD or n(%), unless otherwise stated. Differences were assessed by Student t tests, Mann-Withney U tests or χ2 tests, as appropriate.

|  | **With subclinical CV outcomes (n=17)** | **Without subclinical CV outcomes (n=16)** | **p-value** |
| --- | --- | --- | --- |
| Age, years, mean (range) | 55.67 (21.00 – 77.00) | 57.79 (32.00 – 77.00) | 0.914 |
| Sex, women/men | 7/10 | 6/10 | 0.919 |
|  |  |  |  |
| ***Clinical features*** |  |  |  |
| Albumin, mg/dl | 44.08±2.07 | 36.08±6.78 | 0.625 |
| Urea, mg/dl | 138.27±49.59 | 128.15±39.69 | 0.776 |
| Creatinine, mg/dl | 7.58±1.72 | 8.04±3.51 | 0.724 |
| Plasma Ca, mmol/l | 2.15±0.13 | 2.17±0.18 | 0.905 |
| Plasma phosphate, mmol/l | 1.63±0.45 | 1.66±0.75 | 0.830 |
| PTH, pg/ml | 425.54±219.58 | 309.86±161.53 | 0.220 |
| Total cholesterol, mg/dl | 163.07±40.90 | 155.57±40.84 | 0.482 |
| HDL-cholesterol, mg/dl | 67.29±29.60 | 50.71±15.27 | 0.194 |
| LDL-cholesterol, mg/dl | 74.43±35.66 | 75.00±42.61 | 0.667 |
| Triglycerides, mg/dl | 118.86±72.11 | 144.79±78.90 | 0.265 |
| 25(OH)-vitamin D, ng/ml | 9.69±5.97 | 10.90±7.65 | 0.867 |
| CRP, mg/dl | 0.63±1.11 | 0.52±0.56 | 0.553 |
| Troponin T, ng/l | 66.54±42.55 | 56.79±42.16 | 0.259 |
|  |  |  |  |
| ***Blood cell counts (·10^3^/μl), median (IQR)*** |  |  |  |
| Leukocytes | 6.03 (4.02) | 6.38 (2.95) | 0.847 |
| Neutrophils | 4.08 (1.82) | 3.45 (2.60) | 0.533 |
| Lymphocytes | 1.22 (1.02) | 1.21 (0.76) | 1.000 |
| Monocytes | 0.60 (0.20) | 0.55 (0.21) | 0.234 |
| Eosinophils | 0.23 (0.20) | 0.23 (0.20) | 0.813 |
| Basophils | 0.05 (0.05) | 0.04 (0.05) | 0.400 |
|  |  |  |  |
| ***Clinical features*** |  |  |  |
| Vascular calcifications, n(%) | 9 (59.0) | 9 (52.9) | 0.294 |
| Kauppila score | 8.57±9.34 | 6.31±7.82 | 0.759 |
| Time on dialysis, months | 16.50 (19.00) | 12.00 (15.00) | 0.498 |
| Systolic blood pressure, mm Hg | 131.33±20.55 | 134.42±15.81 | 0.999 |
| Diastolic blood pressure, mm Hg | 77.33±11.23 | 81.85±10.85 | 0.362 |
|  |  |  |  |
| ***Treatments, n(%)*** |  |  |  |
| Paricalcitol | 4 (23.5) | 3 (18.7) | 0.671 |
| Phosphatebinders | 6 (35.2) | 5 (31.2) | 0.611 |
| Statins | 3 (17.6) | 6 (37.5) | 0.201 |
| Metilprednisone | 1 (5.8) | 3 (18.7) | 0.258 |
| Epo | 5 (31.2) | 8 (50.0) | 0.226 |

**Supplementary Table 6: Associations between subclinical CV outcomes and other cell subsets in CKD5-PD patients.** Correlations were assessed by Spearman rank’s tests.

|  | **Number ofneovasa (carotid)** | **Number of neovasa (femoral)** | **aVVarea**  **(cartotid)** | **aVVarea**  **(femoral)** | **PWV** | **cIMT** | **Kauppila** |
| --- | --- | --- | --- | --- | --- | --- | --- |
| **T-cells** |  |  |  |  |  |  |  |
| CD3+ (% lymphocytes) | r=-0.210  p=0.123 | r=0.001  p=0.996 | r=-0.279  p=0.136 | r=-0.214  p=0.085 | r=-0.274  p=0.143 | r=-0.201  p=0.304 | r=-0.280  p=0.091 |
| CD4+ (% CD3+) | r=-0.184  p=0.321 | r=-0.126  p=0.498 | r=0.142  p=0.454 | r=0.070  p=0.706 | r=-0.030  p=0.874 | r=0.105  p=0.597 | r=-0.238  p=0.205 |
| CD8+ (%CD 3+) | r=0.189  p=0.307 | r=0.237  p=0.200 | r=-0.105  p=0.454 | r=0.009  p=0.906 | r=0.079  p=0.677 | r=0.136  p=0.491 | r=0.142  p=0.205 |
| **Leukocytesubsets** |  |  |  |  |  |  |  |
| Leukocytes | r=0.056  p=0.765 | r=0.063  p=0.736 | r=-0.105  p=0.581 | r=-0.036  p=0.849 | r=-0.087  p=0.649 | r=0.032  p=0.872 | r=-0.058  p=0.765 |
| Neutrophils | r=0.051  p=0.784 | r=0.126  p=0.498 | r=0.105  p=0.580 | r=-0.013  p=0.946 | r=0.034  p=0.859 | r=-0.016  p=0.934 | r=-0.088  p=0.651 |
| Lymphocytes | r=-0.062  p=0.741 | r=-0.159  p=0.394 | r=0.125  p=0.511 | r=-0.169  p=0.362 | r=-0.300  p=0.107 | r=-0.036  p=0.855 | r=0.039  p=0.841 |
| Monocytes | r=0.319  p=0.090 | r=0.038  p=0.840 | r=-0.123  p=0.518 | r=0.079  p=0.673 | r=0.285  p=0.127 | r=0.315  p=0.090 | r=0.346  p=0.075 |
| Eosinophils | r=0.000  p=0.999 | r=-0.038  p=0.838 | r=0.307  p=0.099 | r=0.135  p=0.470 | r=0.020  p=0.915 | r=0.274  p=0.158 | r=-0.211  p=0.272 |
| Basophils | r=0.006  p=0.974 | r=-0.055  p=0.769 | r=0.158  p=0.403 | r=0.133  p=0.477 | r=0.076  p=0.689 | r=0.300  p=0.121 | r=-0.118  p=0.541 |

**Supplementary Figure 1: Representative dot-plots from study participants.** Representative dot-plots for study participants (controls and patients) are showed for the different subpopulations analyzed: Tang (A), CD4^+^CD28^null^ (B), monocyte subsets (C) and ACE^+^ (D). Gating of isotype controls are also shown.


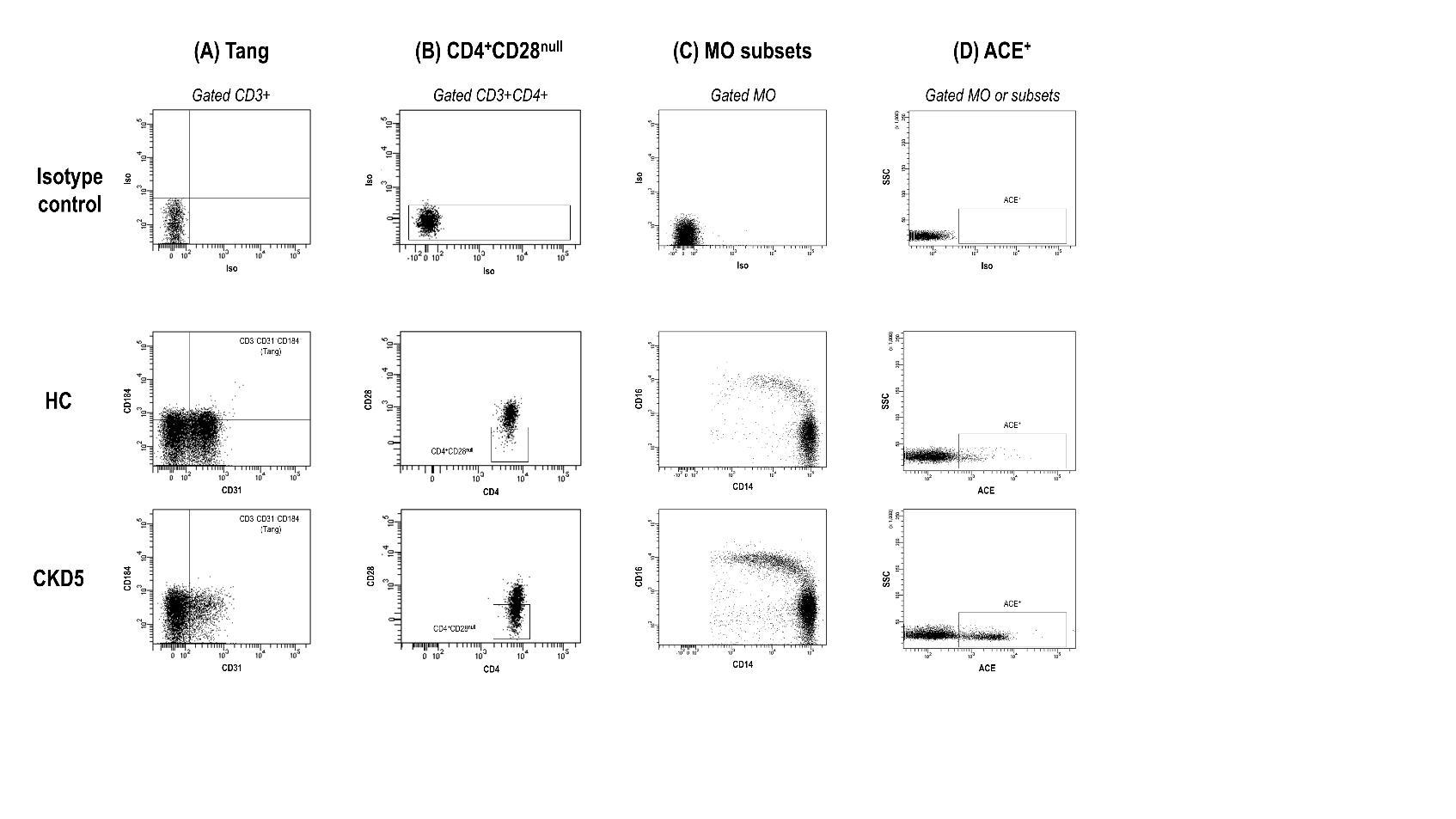


**Supplementary Figure 2: (**A)Representative images for left carotid adventitial Vasa Vasorum (aVV) expression in a healthy controland in a CKD-5PD patient. (B) Identification of adventitial microvessels by an expert radiologist(red lanes).(C) Representative quantification of aVV density using ImageJ.


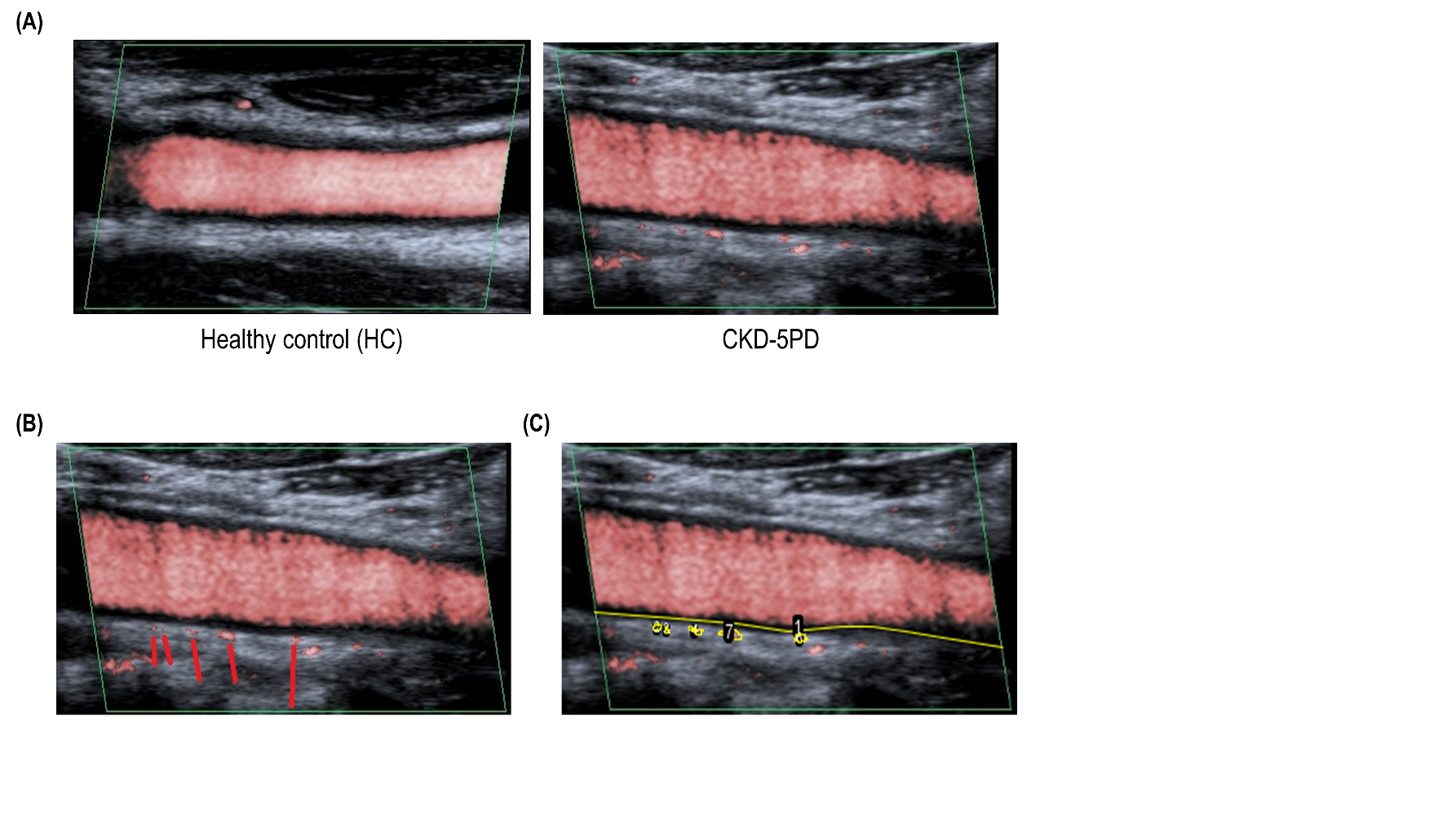

Supplement: Supplementary file 1 [file Data_Sheet_1.docx]
